# Trained-feature–specific offline learning by sleep in an orientation detection task

**DOI:** 10.1167/19.12.12

**Published:** 2019-10-17

**Authors:** Masako Tamaki, Zhiyan Wang, Takeo Watanabe, Yuka Sasaki

**Affiliations:** tamaki@brown.edu; zhiyan_wang@brown.edu; takeo_watanabe@brown.edu; yuka_sasaki@brown.edu; Department of Cognitive, Linguistic, and Psychological Sciences, Brown University, Providence, RI, USA; Department of Cognitive, Linguistic, and Psychological Sciences, Brown University, Providence, RI, USA; Department of Cognitive, Linguistic, and Psychological Sciences, Brown University, Providence, RI, USA; Department of Cognitive, Linguistic, and Psychological Sciences, Brown University, Providence, RI, USA

**Keywords:** *offline learning*, *orientation detection task*, *NREM sleep*

## Abstract

Training-induced performance gains in a visual perceptual learning (VPL) task that take place during sleep are termed “offline performance gains.” Offline performance gains of VPL so far have been reported in the texture discrimination task and other discrimination tasks. This raises the question as to whether offline performance gains on VPL occur exclusively in discrimination tasks. The present study examined whether offline performance gains occur in detection tasks. In Experiment 1, subjects were trained on a Gabor orientation detection task. They were retested after a 12-hr interval, which included either nightly sleep or only wakefulness. Offline performance gains occurred only after sleep on the trained orientation, not on an untrained orientation. In Experiment 2, we tested whether offline performance gains in the detection task occur over a nap using polysomnography. Moreover, we tested whether sigma activity during non-rapid eye movement (NREM) sleep recorded from occipital electrodes, previously implicated in offline performance gains of the texture discrimination task, was associated with the degree of offline performance gains of the Gabor orientation detection task. We replicated offline performance gains on the trained orientation in the detection task over the nap. Sigma activity during NREM sleep was significantly larger in the occipital electrodes relative to control electrodes in correlation with offline performance gains. The results suggest that offline performance gains occur over the sleep period generally in VPL. Moreover, sigma activity in the occipital region during NREM sleep may play an important role in offline performance gains of VPL.

## Introduction

After the initial acquisition of a skill, a learning state goes through an offline process, through which further improvements in performance are obtained without actual training (Karni et al., 1998; Walker, 2005), and these have been termed “offline performance gains.” It has been suggested that sleep plays an essential role in offline performance gains in various types of learning (Bang, Khalilzadeh, Hamalainen, Watanabe, & Sasaki, 2014; Born & Wilhelm, 2012; Diekelmann & Born, 2010; Gais, Molle, Helms, & Born, 2002; Gais, Plihal, Wagner, & Born, 2000; Huber, Ghilardi, Massimini, & Tononi, 2004; Laureys et al., 2001; Maquet et al., 2000; Mascetti et al., 2013; Matarazzo, Franko, Maquet, & Vogels, 2008; McDevitt, Rokem, Silver, & Mednick, 2014; Mednick, Nakayama, & Stickgold, 2003; Rasch & Born, 2013; Stickgold, 2005; Stickgold, James, & Hobson, 2000; Stickgold, Whidbee, Schirmer, Patel, & Hobson, 2000; Tamaki et al., 2013; Tamaki, Matsuoka, Nittono, & Hori, 2008; Tononi & Cirelli, 2014; Walker, 2005; Walker, Brakefield, Morgan, Hobson, & Stickgold, 2002; Yotsumoto, Sasaki, et al., 2009). One type of learning for which sleep is beneficial is visual perceptual learning (VPL; Bang et al., 2014; Censor, Karni, & Sagi, 2006; Gais et al., 2000; Karni, Tanne, Rubenstein, Askenasy, & Sagi, 1994; Mednick et al., 2003; Stickgold, James, & Hobson, 2000; Yotsumoto, Sasaki, et al., 2009). VPL refers to a long-lasting performance improvement on a visual task after visual experience (Lu, Hua, Huang, Zhou, & Dosher, 2011; Sagi, 2011; Sasaki, Nanez, & Watanbe, 2010) and has been proposed to primarily involve reorganization in early visual areas (Schoups, Vogels, Qian, & Orban, 2001; Schwartz, Maquet, & Frith, 2002; Shibata et al., 2017; but see other studies, e.g., Law & Gold, 2008; Xiao et al., 2008). After sleep, either night sleep or a daytime nap, the performance on VPL is significantly enhanced compared to that before sleep. In addition, the amount of improvement after sleep surpasses that after passage of the same amount of time without sleep (Bang et al., 2014; Censor et al., 2006; Gais et al., 2000; Karni et al., 1994; Mednick et al., 2003; Stickgold, James, & Hobson, 2000; Yotsumoto, Sasaki, et al., 2009).

However, it remains to be elucidated whether offline performance gains can be generalized beyond discrimination tasks. The majority of studies that found that sleep plays a role in the performance gains in VPL used the texture discrimination task (TDT), which is a standard task in VPL (Bang et al., 2014; Censor et al., 2006; Gais et al., 2000; Karni et al., 1994; Mednick et al., 2003; Stickgold, James, & Hobson, 2000; Yotsumoto, Sasaki, et al., 2009). Curiously, other studies that found offline performance gains of sleep in VPL also used discrimination tasks, including coarse orientation discrimination (Mascetti et al., 2013; Matarazzo et al., 2008) and motion direction discrimination tasks (McDevitt et al., 2014). The discrimination component of the paradigms in all above-cited studies (Bang et al., 2014; Censor et al., 2006; Gais et al., 2000; Karni et al., 1994; Mascetti et al., 2013; Matarazzo et al., 2008; McDevitt et al., 2014; Mednick et al., 2003; Stickgold, James, & Hobson, 2000; Yotsumoto, Sasaki, et al., 2009b) was not challenging, and involved distinguishing orthogonal orientations of target stimuli or distinguishing opposite motions. Nevertheless, even if the perceptual challenge in these studies may have been driven primarily by the detection component of the task, their paradigms all included a discrimination component. This raises the question whether for offline performance gains of VPL by sleep to occur, a task containing a discrimination component is necessary or not.

An additional question we wanted to address is whether the electrophysiological correlates of offline performance gains would be the same in discrimination and detection tasks. Sleep spindles, which are characteristic spontaneous oscillatory activities during nonrapid eye movement (NREM) sleep, have been suggested to enhance neural plasticity (Rosanova & Ulrich, 2005; Sejnowski & Destexhe, 2000) and have been shown to be involved in declarative and procedural motor memory in humans (Antony et al., 2018; Boutin et al., 2018; Clemens, Fabo, & Halasz, 2006; Fogel & Smith, 2006; Gais et al., 2002; Laventure et al., 2016; Nishida & Walker, 2007; Schabus et al., 2004; Tamaki et al., 2013; Tamminen, Payne, Stickgold, Wamsley, & Gaskell, 2010). Previously, we found that sigma activity (13–16 Hz), whose frequency band corresponds to the sleep spindles source localized in early visual areas (occipital region), was involved in offline performance gains of the TDT (Bang et al., 2014). In the current study, we tested whether the sigma activity recorded from the occipital electrodes is also involved in offline performance gains of the Gabor orientation detection task.

First, the results showed that performance on the Gabor orientation detection task significantly improved after a night of sleep without any additional training. No such offline performance gain was found after the same interval of time that did not include sleep. Second, we found that offline performance gains also occurred after a daytime nap. Moreover, sigma activity was significantly larger in the occipital electrodes than in control electrodes. The performance improvement due to the nap significantly correlated with the power of sigma activity in the occipital electrodes, but not with the control electrodes. The present results suggested that offline performance gains occur not only in discrimination tasks but also in detection tasks and that sigma activity in the trained region of early visual areas during sleep may play a common role in offline performance gains of VPL.

## Methods

### Participants

We conducted a careful screening process for participation eligibility because various factors are known to influence visual sensitivity and sleep stages. All subjects had no prior experience in VPL tasks, as experiences in VPL tasks may cause a long-term visual sensitivity change (Karni & Sagi, 1991; Lu et al., 2011; Sagi, 2011; Sasaki et al., 2010; Schwartz et al., 2002; Seitz et al., 2005; Yotsumoto, Chang, Watanabe, & Sasaki, 2009). People who frequently play action video games were excluded because extensive video game playing affects visual and attention processing (Berard, Cain, Watanabe, & Sasaki, 2015; Green & Bavelier, 2003; Li, Polat, Makou, & Bavelier, 2009). Frequent gamers were defined as those who participated in action video game playing at least 5 hr a week for a continuous period of 6 months or more as defined by previous research (Berard et al., 2015; Green & Bavelier, 2003). In addition, subjects who were included had a regular sleep schedule, i.e., differences in average bedtimes and wake-up times between on weekdays and weekends were less than 2 hr, and the average sleep duration regularly ranged from 6 to 9 hr. Anyone who had a physical or psychiatric disease, was currently under medication, or was suspected to have a sleep disorder was excluded because these factors are known to impact sleep structure (Horikawa, Tamaki, Miyawaki, & Kamitani, 2013; Tamaki, Bang, & Watanabe, 2014; Tamaki, Bang, Watanabe, & Sasaki, 2016). No subjects were suspected of having insomnia, sleep apnea, restless legs syndrome, sleep walking, narcolepsy, or rapid eye movement (REM) sleep behavior disorder, based on a self-reported questionnaire that asked whether a participant had any symptoms known for these sleep disorders. All subjects gave written informed consent for their participation in experiments. This study was approved at the institutional review board at Brown University.

A total of 17 young healthy subjects with normal or corrected-to-normal vision participated in the study. A total of eight subjects participated in Experiment 1. They were randomly assigned to the sleep group (four subjects; three females, 20.0 ± 0.48 years old, *M* ± *SEM*) or to the control wake group (four subjects; three females, 23.0 ± 1.22 years old, *M* ± *SEM*). A total of nine subjects (four females, 24.3 ± 0.55 years old, *M* ± *SEM*) participated in Experiment 2. In Experiment 2, which was conducted using a within-subject design, all subjects had a nap.

### Experimental design for Experiment 1

**Figure 1:**
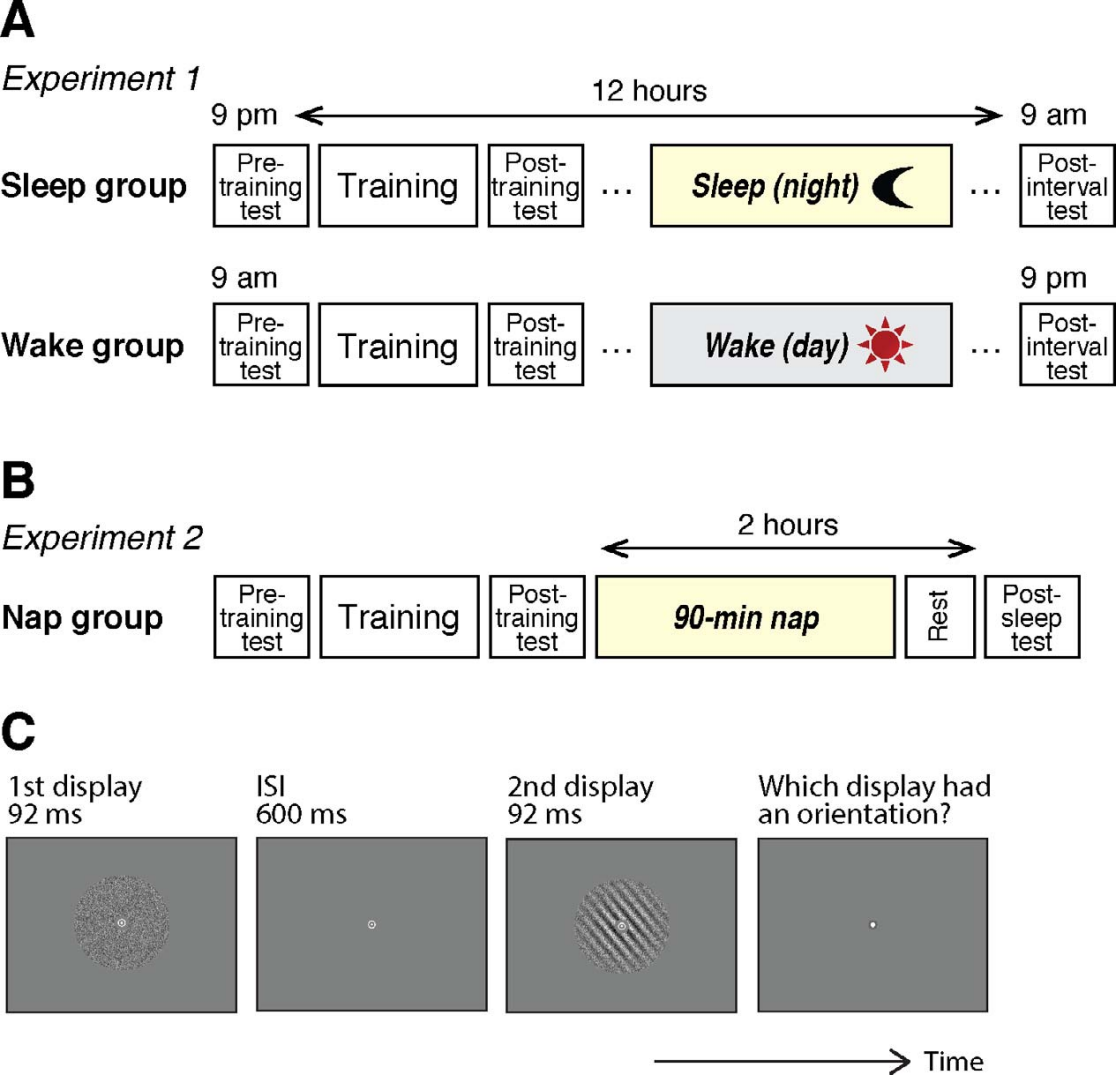
Experimental designs and the Gabor orientation detection task. (A) Experiment 1. (B) Experiment 2. (C) Schematic illustration of one trial of the Gabor orientation detection task.

The subjects in the sleep group arrived at the experimental room at 9 p.m. We explained how to perform the task (the introductory session, see below). Then, the subjects performed a pretraining test session of the orientation detection task (see Orientation detection task below) to measure their initial performance level before training (Figure 1A, Sleep group). After the pretraining test session, which took approximately 5 min, the subjects underwent intensive training on the task for approximately 45 min. After the training session, a posttraining test session (approximately 5 min) was conducted to measure the performance change by training. These three sessions (pretraining test, training, posttraining test) lasted approximately 1 hr in total to complete. There were approximately 2-min breaks between the pretraining test and the onset of training sessions, as well as between the training and the onset of posttraining test sessions. After the completion of the posttraining test session, the subjects slept at their home. The following morning (9 a.m.), the subjects performed a postinterval test session. The postinterval test session lasted approximately 5 min.

The subjects in the wake group arrived at the experimental room at 9 a.m. After the introduction session, they performed a pretraining test session, training session and a posttraining test session, with a 2-min break in between. These sessions took place in the morning between 9 and 10 a.m. (Figure 1A, wake group). At 9 p.m. on the same day, they performed a postinterval test session. No sleep was allowed during the day for the wake group.

The degree of subjective sleepiness was measured by the Stanford Subjective Sleepiness (SSS) scale (Hoddes, Zarcone, Smythe, Phillips, & Dement, 1973) before each of the test sessions for both groups.

### Experimental design for Experiment 2

Subjects in Experiment 2 underwent two sleep sessions at our sleep laboratory: an adaptation and a main experimental sleep session. The adaptation session was conducted prior to the main experimental sleep session for the following reason. It has been shown that when subjects sleep in a sleep laboratory for the first time, the sleep quality is degraded due to the new environment, termed the first-night effect (Agnew, Webb, & Williams, 1966; Carskadon & Dement, 1981; Tamaki et al., 2016; Tamaki, Nittono, Hayashi, & Hori, 2005). To mitigate the first-night effect, an adaptation sleep session was necessary prior to the main experimental sleep session. During the adaptation sleep session, all electrodes were attached to the subjects for polysomnography (PSG) measurement. The subjects slept in the same fashion as in the experimental sleep session. The adaptation session was conducted approximately a week before the main experimental sleep session so that any effects due to sleeping during the adaptation sleep session would not carry over to the experimental sleep session.

On the day of the main experimental sleep session, the subjects arrived at the experimental room approximately at noon (Figure 1B). Then, electrodes were attached for PSG measurement (see PSG measurement), which took approximately 1 hr. After the electrodes were attached, behavioral task sessions were conducted in the same way as in Experiment 1. After the introductory session where we explained how to perform the task, the subjects conducted a pretraining test session of the task to measure their initial performance level before training (∼5 min). After the pretraining test session, the subjects underwent intensive training on the task (∼45 min). After the training session, the posttraining test session (∼5 min) was conducted to measure performance changes by training. There were approximately 2-min short breaks between the pretraining test and training sessions, as well as between the training and posttraining test sessions.

Shortly after the completion of the posttraining test session, room lights were turned off, and the sleep session began at approximately 2 p.m., lasting 90 min. This lights-off time at approximately 2 p.m. was chosen to take advantage of the effect known as the “mid-afternoon dip,” which should facilitate the onset of sleep even in subjects who do not customarily nap (Horikawa et al., 2013; Monk, Buysse, Reynolds, & Kupfer, 1996; Tamaki et al., 2016). During the sleep session, PSG was measured (see PSG measurement). Immediately after the sleep session, a questionnaire was administered to collect data about subjects' introspection regarding the nap (Tamaki et al., 2016). There was a 30-min break after the sleep session to reduce the sleep inertia, which is a prolonged sleepiness upon waking, known to impair performance (Lubin, Hord, Tracy, & Johnson, 1976). After the 30-min break, a postsleep test session (∼5 min) was conducted to measure the changes in performance over the sleep session.

Subjective (SSS; Hoddes et al., 1973) and behavioral sleepiness (PVT; Dinges & Powell, 1985) were measured three times prior to each test session (see Sleepiness measurement).

To collect data about subjects' sleep habits and handedness, three types of questionnaires were administered prior to an adaptation sleep session. They were the Pittsburg Sleep Quality Index questionnaire (PSQI; Buysse, Reynolds, Monk, Berman, & Kupfer, 1989), the Morningness-Eveningness Questionnaire (MEQ; Horne & Ostberg, 1976), and the Edinburgh Handedness Questionnaire (Oldfield, 1971). Using the PSQI, we measured the following parameters: habitual sleep quality (%; calculated as [number of hours slept / number of hours spent in bed] × 100), average bedtime, wake-up time, subjective sleep-onset latency, and global PSQI score. The global PSQI score indicates the quality of subjects' habitual sleep (range: 0–21, a global score of >5 suggests poor sleep). The MEQ estimates individual circadian variations. All subjects in Experiment 2 were right-handed according to the answers on the handedness questionnaire.

### Orientation detection task

A Gabor patch was used for the orientation detection task (Figure 1C). The diameter of the Gabor patch was 10°, presented at the center of the screen. The spatial frequency of the Gabor patch was 1 cycle per degree, and the Gaussian filter sigma was 2.5°. Gabor patches were spatially masked by a noise pattern that was generated from a sinusoidal luminance distribution at a given signal-to-noise ratio (SNR; Shibata et al., 2017). The average luminance of the stimulus was 130.7 ± 3.28 cd/m^2^. The orientation of the Gabor patch was 10°, 70°, or 130°. One orientation was randomly selected as the trained orientation for each subject. Another orientation was randomly selected as an untrained orientation. The remaining orientation was used for the introductory session (see below).

Subjects performed the orientation detection task with a two-interval forced choice (2IFC) as in previous studies (Shibata et al., 2017; Xiao et al., 2008). Subjects were presented with two types of displays. One display contained a Gaussian windowed sinusoidal grating (Gabor) patch with a certain SNR. The other display had only noise (0% SNR). Each trial started with a 500-ms fixation interval. Two displays were presented sequentially for 92 ms, with a 600-ms interstimulus-interval (Xiao et al., 2008; Zhang et al., 2010). The temporal order of the two displays was randomly determined in each trial. After the two displays were presented, subjects were asked to report which display (the first or the second) contained stripes by pressing the “1” or “2” button on a keyboard. No feedback on the accuracy of a response was provided. Subjects were instructed to fixate on a white bull's eye fixation point (diameter = 1.5°) throughout the display presentations for each trial.

The test sessions measured the threshold SNR for the trained and untrained orientations, for each of which one block of staircase was performed. The initial SNR was set to 25%. The step size of the staircase was 0.03 log units to adjust the SNR with a two-down, one-up staircase procedure, which yields approximately 70% accuracy. Each block ended after 10 reversals, which resulted in approximately 40 trials per orientation. The geometric mean of the last six reversals in each block was obtained as the threshold SNR for the orientation.

In the training session, a total of 600 trials were performed in six blocks with the trained orientation. The number of trials per block for the training session was fixed at 100 trials. The initial SNR and the step size of the two-down, one-up staircase procedure were the same as the test sessions.

The performance improvement (%) by training was calculated as the percent change in the threshold SNR measured at the posttraining test session normalized by that at the pretraining test session (performance improvement at posttraining: [pretraining – post-training]/pretraining × 100). The performance improvement (%) by the interval (sleep or wake) was calculated at the postinterval test session relative to the posttraining test session (performance improvement at postinterval: [posttraining – postinterval]/posttraining × 100).

During the introductory session, we explained how to perform the 2IFC Gabor orientation detection task before the pretraining test in both Experiments 1 and 2. There were approximately 20–30 trials in this session until the SNR was below 0.1. The orientation of the Gabor used for this session was neither trained nor untrained orientation, as mentioned above. In addition, just before the postinterval test session in Experiment 1 and just before the postsleep session in Experiment 2, the second introductory session was performed with approximately 10 trials to remind the subjects of what the task was.

### PSG measurement

In Experiment 2, the attachment of electrodes for polysomnogram (PSG) measurement, which took approximately 45 min, was conducted prior to the first introductory session of the orientation detection task. PSG consisted of EEG, electrooculogram (EOG), electromyogram (EMG), and electrocardiogram (ECG). EEG was recorded at 25 scalp sites according to the 10% electrode position (Sharbrough, Lesser, Lüders, Nuwer, & Picton, 1991) using active electrodes (actiCap, Brain Products, LLC) with a standard amplifier (BrainAmp Standard, Brain Products, LLC). The online reference was Fz, and it was re-referenced to the average of the left (TP9) and right (TP10) mastoids after the recording. The sampling frequency was 500 Hz. The impedance of the active electrodes was kept below 20 kΩ. The active electrodes included a new type of integrated impedance converter, which allowed them to transmit the EEG signal with significantly lower levels of noise than traditional passive electrode systems. The data quality with active electrodes with impedance below 20 kΩ was as good as 5 kΩ using passive electrodes (Tamaki et al., 2016). The passive electrodes were used for EOG, EMG, and ECG (BrainAmp ExG; Brain Products, GmbH, Gilching, Germany). Horizontal EOG was recorded using two electrodes placed at the outer canthi of both eyes. Vertical EOG was measured using four electrodes 3 cm above and below both eyes. EMG was recorded from the mentum (chin). The impedance was maintained at approximately 10 kΩ for the passive electrodes. Brain Vision Recorder software (Brain Products, LLC) was used for recording. The data were filtered between 0.1 and 100 Hz. PSG was recorded in a soundproof and shielded room.

### Sleep-stage scoring and sleep parameters

Based on the PSG data acquired during Experiment 2, sleep stages were scored for every 30-s epoch, following standard criteria (Iber, Ancoli-Israel, Chesson, & Quan, 2007; Rechtschaffen & Kales, 1968), as stage wakefulness (stage W), NREM stage 1 sleep (stage N1), NREM stage 2 sleep (stage N2), NREM stage 3 sleep (stage N3), and stage REM sleep (REM sleep). Standard sleep parameters were obtained to indicate a general sleep structure for each experiment to confirm that there is no abnormality in subjects' sleep structures. Sleep parameters included the sleep-onset latency (SOL; the latency to the first appearance of stage N2 from the lights-off), the percentage of each sleep stage, wake time after sleep onset (WASO; the total time of stage W after sleep onset), sleep efficiency (SE; the total percentage spent in sleep), and the time in bed (TIB; the time interval between lights-off and lights-on; Iber et al., 2007).

### EEG analyses

A fast-Fourier transformation was applied to the EEG data in 5-sec epochs and smoothed with a tapered cosine window (Nobili et al., 1999) to compute brain activities. Six epochs were used to yield the averaged spectral data of 30 s. Spectral power for sigma activity (13–16 Hz frequency band) was obtained during NREM sleep stages (from both N2 and N3). Sigma activity in the occipital region was obtained by averaging sigma power measured across six occipital electrodes (PO3, PO7, O1, PO4, PO8, O2) that would roughly reflect activity in early visual areas (Censor, Bonneh, Arieli, & Sagi, 2009; Polat, Sterkin, & Yehezkel, 2007; Sterkin, Yehezkel, Bonneh, Norcia, & Polat, 2008; Sterkin, Yehezkel, & Polat, 2012; Thut, Nietzel, Brandt, & Pascual-Leone, 2006; Viemose et al., 2013), which are assumed to be involved in the Gabor orientation detection task based on previous studies. We also obtained sigma activity from control electrodes (P7, P8) that are close to the middle temporal gyrus (or MT area; Seeck et al., 2017). The MT area was chosen as a control because this region is unlikely to play a critical role in offline performance gains of a Gabor orientation detection task. We computed region-specific sigma activity by subtracting the sigma activity of the control electrodes from that of the occipital electrodes.

### Statistical analyses

The α level (Type I error rate) of 0.05 was set for all statistical analyses. The Shapiro-Wilk test, which can be used for small sample sizes (Shapiro & Wilk, 1965), was conducted for all the data, by which we confirmed that all the data were normally distributed (all *p*s > 0.05). Levene's test was conducted to test for homogeneity of variance. It was confirmed that homogeneity of variance was not violated for all the data (all *p*s > 0.05). We conducted the Grubbs test, by which no outlier data were detected.

To analyze performance improvement, ANOVA was first conducted, and *t* tests were conducted as post hoc tests in Experiment 1. In Experiment 2, we conducted *t* tests on performance improvement and sigma activity. When a correction for multiple comparisons was necessary for multiple *t* tests, we controlled the false discovery rate (FDR; Benjamini & Hochberg, 1995) to be at 0.05. To obtain correlation coefficients, Pearson's correlation was used.

Statistical tests were conducted using SPSS (ver. 22, IBM Corp.).

## Results

### Experiment 1

We hypothesized that offline sleep-dependent performance gains would occur in the Gabor orientation detection task. If this was the case, then the performance improvement over the interval should be larger in the sleep group than in the wake group. In addition, because the Gabor orientation detection task has trained-feature specificity (Shibata et al., 2017), we hypothesized that sleep-dependent offline performance gains occur with the trained orientation, not with the untrained orientation.

**Figure 2:**
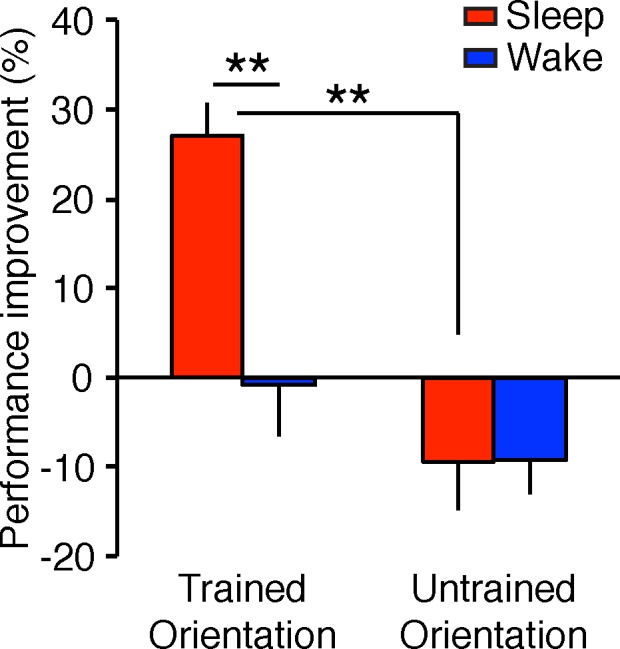
The mean performance improvement (± SEM) at the postinterval test session for the sleep and the wake groups in Experiment 1. See the main text for the results of ANOVA. Asterisks (**) indicate that post hoc t tests (one sample t test and a paired t test) showed significance at p < 0.01. An FDR correction was applied (see the main text for details).

To test whether performance improvement over the interval was larger in the sleep condition and whether the improvement was specific to the trained orientation, we conducted a 2-way mixed-design ANOVA with group (sleep vs. wake) and orientation (trained vs. untrained) factors on the performance improvement at the postinterval test session. The results are shown in Figure 2. ANOVA indicated a significant two-way interaction, *F*(1, 6) = 6.51, *p* = 0.043; a significant main effect of group, *F*(1, 6) = 12.63, *p* = 0.012; and a significant main effect of orientation, *F*(1, 6) = 16.45, *p* = 0.007. For the trained orientation, the post hoc tests revealed that there was a significant difference in the performance improvement between the sleep and wake groups but not for the untrained orientation (trained, *t*[6] = 4.11, *p* = 0.006, *q* < 0.05, FDR for two comparisons; untrained, *t*[6] = 0.04, *p* = 0.967). Furthermore, one-sample *t* tests showed that the performance improvement for the trained orientation at the postinterval session was significantly different from 0 for the sleep group but not for the wake group (the sleep group, *t*[3] = 7.49, *p* = 0.005, *q* < 0.05, FDR for four comparisons including the following three 1-sample *t* tests; the wake group, *t*[3] = 0.14, *p* = 0.897). In contrast, the performance improvement for the untrained orientation at postinterval was not significantly different from 0 for both groups (one-sample *t* test, sleep group: *t*[3] = 1.74, *p* = 0.181; wake group: *t*[3] = 2.29, *p* = 0.106). Thus, offline performance gains were found for the Gabor orientation detection task, and this occurred only with the trained orientation. These results were consistent with the hypotheses.

We performed the following control analyses to rule out the possibility that the difference in offline performance between the groups was caused by factors other than the experimental manipulation.

First, we tested whether the initial SNR threshold was different before training between the groups. However, we did not find a significant difference between the sleep and wake groups in the SNR threshold in the pretraining test session, *t*(6) = 0.07, *p* = 0.945.

Next, we tested whether the training effect was different between the sleep and wake groups. A two-way mixed model ANOVA with group (sleep vs. wake) and orientation (trained vs. untrained) factors was conducted on the performance improvement at the posttraining test session. However, there was no significant main effect of group, *F*(1, 6) = 0.06, *p* = 0.811; no significant main effect of orientation, *F*(1, 6) = 0.02, *p* = 0.899; or no significant interaction between the factors, *F*(1, 6) = 0.48, *p* = 0.514. The results indicated that the effect of training was not significantly different between the groups.

Third, we confirmed that the threshold SNR for the training session was not significantly different between the groups (threshold SNR: sleep group, 0.04 ± 0.003; wake group, 0.05 ± 0.004; unpaired *t* test, *t*[6] = 1.96, *p* = 0.101).

Finally, we tested whether the degree of subjective sleepiness was different between the sleep and the wake groups, as the sleepiness may impact the performance of the detection task. A two-way mixed design ANOVA with group (sleep vs. wake) and session (pretraining, posttraining, postinterval) was conducted on the SSS ratings. The main effect of group, *F*(1, 6) = 0.17, *p* = 0.695; the main effect of session, *F*(2, 12) = 3.17, *p* = 0.079; or interaction between the factors, *F*(2, 12) = 1.50, *p* = 0.262, were not significant.

These results indicated that the significant difference between the two groups in the performance improvement over the interval at the postinterval test session cannot be attributed to the initial performance, the effect of training, or subjective sleepiness.

### Experiment 2

In Experiment 2, we tested whether sleep-dependent offline performance gains occur with a nap in the Gabor orientation detection task and investigated whether sigma activity during NREM sleep was involved in sleep-dependent offline performance gains of the task.

#### VPL performance

**Figure 3:**
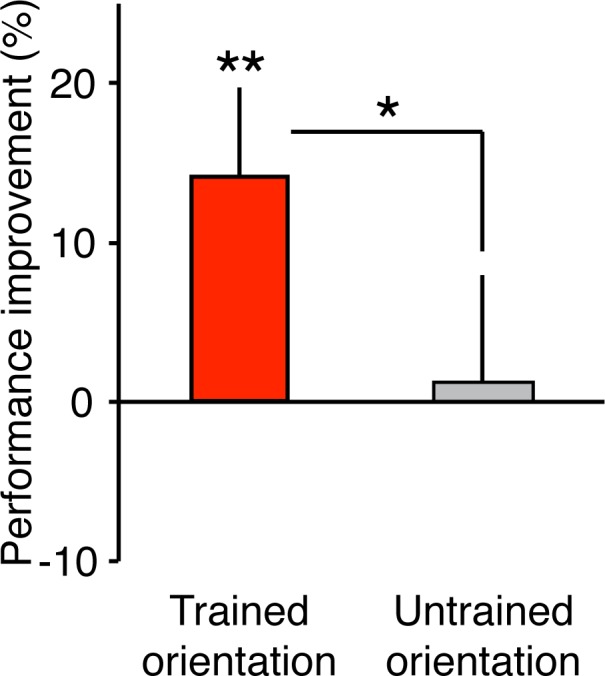
The mean performance improvement (± SEM) at the post interval test session for Experiment 2. N = 9. Paired and one-sample t tests, **p < 0.01; *p < 0.05. See the main text for additional details of the statistical results.

We first examined whether the performance on the orientation detection task was improved only in the trained orientation after a daytime nap. We conducted one-sample *t* tests for the performance changes for each of the trained and untrained orientations at the post-sleep test session. The results indicated that sleep-dependent offline performance gains occurred with the nap in the trained orientation (Figure 3). The performance was significantly improved for the trained orientation but not for the untrained orientation (trained, *t*[8] = 3.55, *p* = 0.008, *q* < 0.05, FDR for two comparisons; untrained, *t*[8] = 0.25, *p* = 0.811). Furthermore, a paired *t* test showed that the performance improvement at postsleep was significantly different between the trained and untrained orientations, *t*(8) = 2.79, *p* = 0.024. Thus, the offline performance gains produced by the nap were specific to the trained orientation.

Importantly, the difference in the performance improvement between the trained and untrained orientations was not apparent until after sleep. First, there was no significant difference in the initial SNR threshold between the trained versus untrained orientations at the pretraining test session before training (trained: 0.046 ± 0.003, untrained: 0.043 ± 0.004; *t*[8] = 1.29, *p* = 0.234). Second, there was no significant difference in the performance improvement (%) by training at the posttraining test session, *t*(8) = 0.65, *p* = 0.531. These statistical tests indicate that the performance improvement specific to the trained orientation emerged only after sleep.

We compared the amount of offline performance gains obtained in Experiments 1 and 2. There was no significant difference in the amount of offline performance gains between the experiments, *t*(15) = 0.14, *p* = 0.887. Thus, offline performance gains induced by sleep at night and by a nap did not significantly differ.

#### Sigma activity during NREM sleep and offline performance gain

**Figure 4:**
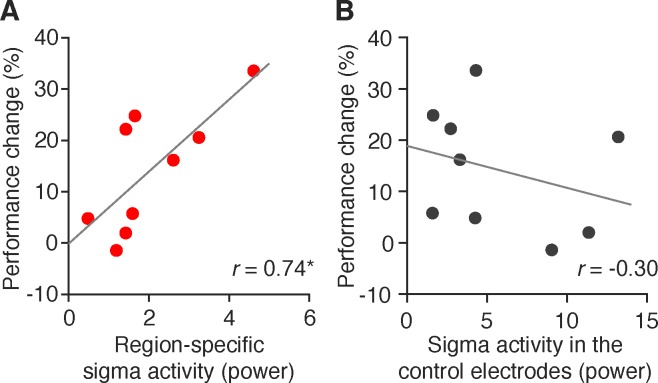
Correlation between offline performance gains and (A) region-specific sigma activity and (B) sigma activity in the control electrodes during NREM sleep. N = 9. *p < 0.05. No outliers were detected by Grubbs' test.

We tested the hypothesis that sigma activity was involved in the offline performance gain of the detection task in a region-specific manner. First, we obtained sigma activity in the occipital and control electrodes (see EEG analyses in Methods for more details). We tested whether sigma activity was larger in the occipital relative to the control electrodes. We found a significant difference in sigma activity between the occipital and control electrodes (paired *t* test, *t*[8] = 4.86, *p* = 0.001). Sigma activity was significantly larger in the occipital than in the control electrodes. Next, we tested whether the region-specific sigma activity, which was obtained by subtracting the sigma activity of the control electrodes from that of the occipital electrodes, was correlated with the performance change over a period of sleep (from the posttraining test session to the postsleep test session; see Table 1 for sleep parameters for the sleep session). We found a significant correlation between them (Figure 4A; *r* = 0.74, *p* = 0.024, *n* = 9, *q* < 0.05, FDR for two comparisons). The Grubbs' test indicated that there were no outliers in the data. We conducted a control test to examine whether there was a similar correlation between the performance change and sigma activity obtained from the control electrodes. We did not find a significant correlation between them (Figure 4B; *r* = −0.30, *p* = 0.438). Thus, the result was consistent with the hypothesis that sigma activity was involved in the offline performance gain of the detection task in a region-specific manner.

**Table 1:** Sleep parameters for Experiment 2. Notes: SOL = sleep-onset latency; WASO = wake time after sleep onset; SE = sleep efficiency; TIB = the time in bed which indicates the duration of each sleep session (the time interval between lights-off and lights-on).

	*M* ± *SEM*
Stage W (min)	21.4 ± 5.61
Stage N1 (min)	11.2 ± 2.45
Stage N2 (min)	25.3 ± 4.03
Stage N3 (min)	18.0 ± 5.30
REM sleep (min)	5.4 ± 2.61
SOL (min)	11.0 ± 3.84
WASO (min)	14.2 ± 4.76
SE (%)	73.6 ± 7.45
TIB (min)	85.2 ± 1.96

We next tested whether any macroscopic sleep variables, such as the duration of each sleep stage, were associated with the offline performance gain (see Table 1 for all the sleep parameters). We determined the Pearson's correlation coefficient with the performance change over the sleep period and the durations of each of the sleep stages (W, N1, N2, N3, REM sleep). We found that none of the durations of sleep stages were significantly correlated with offline performance gain (stage W, *r* = −0.18, *p* = 0.642; stage N1, *r* = 0.31, *p* = 0.418; stage N2, *r* = 0.10, *p* = 0.791; stage N3, *r* = −0.25, *p* = 0.517; REM sleep, *r* = 0.49, *p* = 0.181; no corrections for multiple comparisons).

We calculated other sleep parameters, such as SOL (from the lights-off to sleep onset time [min]), SE (the total sleep time divided by the time in bed × 100 [%]),WASO (min), and TIB (min), as shown in Table 1. We tested whether these parameters explain the offline performance gains by calculating correlation coefficients. However, none of them was significantly correlated with offline performance gains (SOL, *r* = 0.23, *p* = 0.551; SE, *r* = 0.18, *p* = 0.645; WASO, *r* = −0.35, *p* = 0.350; TIB, *r* = 0.17, *p* = 0.669).

#### Quality of habitual sleep and circadian variations

We next examined whether any of the measures of sleep habits or habitual sleep quality were related to the performance change. We analyzed the quality of subjects' habitual sleep using the PSQI questionnaire (Buysse et al., 1989) and whether they were extreme morning or evening types determined by the MEQ questionnaire (Horne & Ostberg, 1976). The PSQI and MEQ were administered prior to sleep sessions in Experiment 2 (Table 2).

**Table 2:** Habitual sleep parameters. Notes: PSQI = Pittsburg Sleep Quality Index questionnaire; MEQ = Morningness-Eveningness Questionnaire.

	*M* ± *SEM*
Bedtime	23:16 ± 0:09
Wake-up time	7:42 ± 0:16
Sleep duration	7.80 ± 0.20
Sleep onset latency	11.90 ± 2.57
Habitual sleep efficiency (%)	92.80 ± 2.25
Global PSQI score	2.70 ± 0.37
MEQ score	55.20 ± 2.63

The PSQI assesses whether subjects have any sleep problems (Buysse et al 1989). If the global PSQI score is 5 or higher, this suggests that subjects have a sleep problem. However, the average global PSQI score in the subjects of Experiment 2 was 2.7 ± 0.37 and hence fell within a 1–4 range, which indicated that none of the subjects were suspected of having sleep problems. In addition, based on the PSQI data, we obtained the subjects' habitual bedtime, wake-up time, average sleep-onset latency, estimated sleep duration, and habitual sleep efficiency (Table 2). These scores in the present subjects in Experiment 2 fell within a normal range (Buysse et al., 1989).

The MEQ assesses whether the subject is a morning type or evening type (Horne & Ostberg, 1976). Such variations in the circadian timing may affect performance (Kerkhof, 1985). The average MEQ score was 55.2 ± 2.63 with a range between 46–67, which fell into the intermediate type of morningness-eveningness (neither extreme morning nor evening type; Horne & Ostberg, 1976). These results confirmed that none of the subjects had sleep problems or could be categorized into extreme morning or evening type, suggesting that all the subjects had normal sleep-wake habits and were all good sleepers.

Then, we examined whether any of the measures of sleep habits or habitual sleep quality were related to the performance change. We determined the Pearson's correlation coefficient between offline performance gain over the sleep period and habitual sleep efficiency, habitual sleep duration, global PSQI score, and MEQ score. None of these showed a significant correlation with offline performance gain (habitual sleep duration, *r* = 0.54, *p* = 0.134; habitual sleep efficiency, *r* = 0.52, *p* = 0.150; global PSQI score, *r* = 0.12, *p* = 0.763; MEQ score, *r* = −0.66, *p* = 0.052; no correction for multiple comparisons).

## Discussion

The present results demonstrated feature-specific offline performance gains in a Gabor orientation detection task, during both nocturnal and daytime sleep. Previous studies that tested offline performance gains in VPL used tasks with a discrimination component. Thus, it was unclear whether offline performance gains of VPL by sleep were specific to paradigms including a discrimination component. However, our results indicated that offline performance gains in VPL by sleep also occur with a pure detection task and that they are therefore independent from the nature of the task.

In addition, we found that regional sigma activity recorded from occipital electrodes was significantly correlated with performance changes on the orientation detection task between before and after sleep. It had been shown that sigma activity was involved in the offline performance gain in a different task (TDT) over the sleep period (Bang et al., 2014). Thus, it is possible that offline performance gain is commonly associated with regional sigma activity during sleep in visual areas for VPL. Sigma activity corresponds to the activity of sleep spindles, which are associated with various types of learning, including learning on declarative and procedural motor tasks (Antony et al., 2018; Boutin et al., 2018; Clemens et al., 2006; Fogel & Smith, 2006; Gais et al., 2002; Laventure et al., 2016; Nishida & Walker, 2007; Schabus et al., 2004; Tamaki et al., 2013; Tamminen et al., 2010). Sleep spindles are specific waves used for sleep stage scoring and appear around the central electrode locations during NREM sleep (Iber et al., 2007; Rechtschaffen & Kales, 1968). In the present study, because early visual areas were the targeted regions for offline performance gains of VPL over the sleep period, we extracted the strength of sigma activity that corresponded to the frequency of sleep spindles from the occipital electrodes. The precise causal relationship between sigma activity and learning has yet to be clarified. However, it has been shown that stimulation at a frequency that corresponds to sleep spindles, increases long-term potentiation (Rosanova & Ulrich, 2005) and that modulations of sleep spindles or relevant spontaneous brain activities influence learning (Girardeau, Benchenane, Wiener, Buzsaki, & Zugaro, 2009; Novitskaya, Sara, Logothetis, & Eschenko, 2016). Thus, the present results support the idea that regional sigma activity in early visual areas plays a crucial role in offline performance gains of VPL, possibly by increasing regional plasticity in early visual areas during NREM sleep.

However, the spatial resolution of EEG activity is limited in our study, as the strength of sigma activity was obtained from the occipital electrodes without any source-localization techniques. Thus, the sources of EEG activity could be spread so that the current results that seem to support the hypothesis may be due to very different sources of activity from what we assumed. However, we argue that the EEG activity from the occipital region in the present study may represent activity of early visual areas based on the following reasons. First, previous studies have shown that the EEG activity in the occipital region is closely matched to blood-oxygen level dependent (BOLD) signals of early visual areas using functional magnetic resonance imaging that has high spatial resolution (Scheeringa, Fries, et al., 2011; Scheeringa, Mazaheri, Bojak, Norris, & Kleinschmidt, 2011). Additionally, visual processing that is known to occur in early visual areas was reflected in the event-related potentials of the occipital electrodes (Censor et al., 2009; Polat et al., 2007; Sterkin et al., 2008). The event-related potential of the occipital electrodes was source-localized to early visual areas (Proverbio, Del Zotto, & Zani, 2010). Second, we found that the region-specific activity, in which activity in the control electrodes was subtracted from that in the occipital electrodes, was significantly correlated with offline performance gains, whereas the sigma activity from the control electrodes alone was not. This indicates that the region-specific activity did not spread widely to reach control electrodes. These suggest that sigma activity recorded from the occipital electrodes at least partially reflected activities in early visual areas that are involved in sleep-dependent offline performance gains of VPL.

It is important to note that the quality of sleep was not correlated with the performance improvement over the sleep period in Experiment 2. Of all the sleep quality measures we took (sleep efficiency, wake time after sleep onset, sleep-onset latency and time in bed, PSQI score, MEQ score), none was correlated with offline performance gain. If the wake time during the sleep session was the cause of offline performance gains, there should have been a good correlation for each parameter with offline performance gains. In addition, the results of Experiment 1 clearly showed that the interval that contained sleep led to performance improvements, while the interval that included only wakefulness did not lead to performance improvements. These results demonstrate that the offline performance gain of an orientation detection task is a sleep-state dependent process.

The present study focused on the role of sleep—in particular, sigma activity during NREM sleep—on performance in a detection task. However, we do not deny the possibility that REM sleep plays a critical role in VPL, as has been indicated by a previous study (Karni et al., 1994). In addition, the present study investigated the enhancement of performance by sleep. However, some studies have suggested that sleep plays a role in rendering learning, which was trained before sleep, resilient to new learning that occurs after sleep by reducing retrograde interference (Albouy et al., 2016; Ellenbogen, Hulbert, Stickgold, Dinges, & Thompson-Schill, 2006; Fishbein, 1971; Korman et al., 2007; Linden, Bern, & Fishbein, 1975; Sonni & Spencer, 2015). This suggests that sleep contributes to not only enhancing but also stabilizing learning. Which sleep stages or brain oscillations are involved in stabilizing VPL needs to be clarified in future research.

## Conclusions

In conclusion, the present study demonstrates that offline performance gains during sleep were found on an orientation detection task specifically to the trained orientation. This indicates that offline performance gains are not limited to discrimination tasks including TDT. The present results also suggest that regional sigma activity contributes to the facilitation of VPL during sleep.
